# PECTOPLATE: the simultaneous phenotyping of pectin methylesterases, pectinases, and oligogalacturonides in plants during biotic stresses

**DOI:** 10.3389/fpls.2015.00331

**Published:** 2015-05-13

**Authors:** Vincenzo Lionetti

**Affiliations:** Dipartimento di Biologia e Biotecnologie Charles Darwin, Sapienza Università di Roma, Rome, Italy

**Keywords:** pectin methylesterase, pectin methylesterase inhibitor, pectinase, *Botrytis*, oligogalacturonides, PECTOPLATE, *Arabidopsis*

## Abstract

Degradation of pectin, a major component of plant cell wall, is important for fungal necrotrophs to achieve a successful infection. The activities of pectin methylesterases (PMEs) from both plants and pathogens and the degree and pattern of pectin methylesterification are critical for the outcome of plant–pathogen interaction. Partial degradation of pectin by pectin degrading enzymes releases oligogalacturonides (OGs), elicitors of plant defense responses. Few analytical techniques are available to monitor pectin methylesterification-modulating machineries and OGs produced during plant pathogen interaction. In the present study, ruthenium red is presented as useful dye to monitor both *Botrytis cinerea* mycelium growth and the induction of PME activity in plant tissue during fungal infection. Moreover a simple, inexpensive and sensitive method, named PECTOPLATE, is proposed that allows a simultaneous phenotyping of PME and pectinase activities expressed during pathogen infection and of pectinase potential in generating OGs. The results in the manuscript also indicate that PME inhibitors can be used in PECTOPLATE as a tool to discriminate the activities of plant PMEs from those of pathogen PMEs expressed during pathogenesis.

## Introduction

The cell wall (CW) represents a barrier that pathogens need to breach to colonize the plant tissue. Emerging evidence points out that dynamic changes in the composition and structure of the CW during infection are exploited by both plant and pathogen to prevail during their interaction (Bellincampi et al., 2014; Lionetti and Metraux, 2014). Degradation of pectin, a major component of CW, is important for fungal necrotrophs to achieve a successful infection (ten Have et al., 1998; Kars et al., 2005). The activity of pectic degrading enzymes is required to disassemble the CW matrix and to expose the diverse CW polymers to the action of other CW degrading enzymes (Pogorelko et al., 2013a,b). Pectin is synthesized in the Golgi and secreted in a highly methyl esterified form into the CW. In this compartment, pectin methylesterases (PMEs, E.C. 3.1.1.11; Pfam 01095; CE8, www.cazy.org), catalyze the de-methylesterification of pectin releasing free carboxyl ester groups, protons and methanol.

The activities of PMEs from both plants and pathogens and the degree and pattern of pectin methylesterification are critical for the outcome of plant–pathogen infections (Lionetti et al., 2012). Plants finely tune pectin methylesterification and regulate PME activities through endogenous inhibitors indicated as PME inhibitors (PMEIs; Raiola et al., 2004; Lionetti et al., 2012; Reca et al., 2012). Using genetic, biochemical and molecular approaches it has been shown that the overexpression of PMEIs in *Arabidopsis*, wheat, pepper and tobacco results in a reduced susceptibility to fungal, bacterial and viral pathogens (Lionetti et al., 2007, 2014, 2015b; An et al., 2008; Raiola et al., 2011; Volpi et al., 2011). The higher methylesterification detected in these plants makes pectin less susceptible to hydrolysis by microbial pectinases and, consequently, microbial growth is reduced. Methylesterification of pectin could be modulated by plant or pathogens during the infection process. Specific plant PMEs can be recruited by fungal pathogens to prevail during infection. AtPME3, an *Arabidopsis* PME isoform, is induced upon infection with *Botrytis cinerea* and *Pectobacterium carotovorum* and is necessary for a successful infection (Raiola et al., 2011). In a recent paper, interesting correlations have been found in wheat cultivars among a low level of expression of a PME gene WheatPME1, a high degree of methylesterification, a low random distribution of methylesterification and an high resistance to *Fusarium graminearum* (Lionetti et al., 2015a). Jasmonic acid has been proposed to modulate the degree of methylesterification in potato to protect pectin degradation by pectate lyase produced by *Dickeya dadantii* (Taurino et al., 2014). *Arabidopsis* PME activity is triggered through a JA-dependent pathway during *Arabidopsis* infection with the necrotrophic fungus *Alternaria brassicicola* (Bethke et al., 2014). The extensive degradation of homogalacturonan (HG) by pathogens is sensed by plants. HG breakdown fragments [e.g., oligogalacturonides (OGs)], released upon partial degradation of HG by fungal PGs, are the best characterized damage-associated molecular patterns in plants (Ferrari et al., 2008, 2013). Some evidence indicates that PME activity can influence elicitor activity of OGs (Osorio et al., 2008, 2011).

It is now clear that the modulation of pectin methylesterification by plant and microbial PMEs are critical for the outcome of plant diseases but the current knowledge about the factors governing these processes is limited. Few efforts have been devoted so far to develop methods highlighting the mechanisms that underline plant–microbe interactions. Therefore, new analytical techniques are needed to study the role of plant and pathogen CW modifying enzymes during infection, and in particular, to gain deeper insight on the function of pectin methylesterification-modulating machineries during plant–pathogen interaction. Ruthenium red (RR) is a cationic dye with six positive charges, capable to forms electrostatic bonds with the acidic groups of polyuronic acids of pectin (Sterling, 1970; Luft, 1971). The binding of RR to pectin increases as the degree of methylesterification decreases. Differences in RR affinity for methylesterified and de-esterified pectin have been previously used to develop a specific and sensitive gel diffusion enzyme assay for the determination of purified PME and PG activities (Mckay, 1988; Cotty et al., 1990; Downie et al., 1998) and for a fast quantitation of PME activity in crude plant extracts (Downie et al., 1998; Lionetti et al., 2007).

The present study reveals RR staining as a useful tool to monitor both *B. cinerea* mycelium growth and induction of PME activity in plant tissue during fungal infection. Moreover, a new methodology, named PECTOPLATE, is proposed as a tool for the simultaneous phenotyping of PME and pectinase activities expressed in plant tissues during pathogen infection and of their ability to generate OGs. The results also indicate that PMEIs can be used in PECTOPLATE to discriminate plant PMEs activities from those expressed by pathogen during infection.

## Materials and Methods

### *Arabidopsis* Infection with *B. cinerea*

*Botrytis cinerea* strain SF1 (Lionetti et al., 2007) was grown for 15 days on potato dextrose agar (PDA) at 39 g L^–1^ at 23°C with a 12-h photoperiod before spores collection. The spores were harvested by washing the surface of the mycelium with 10 mL of sterile distilled water. Spore suspensions were filtered through glass wool to remove residual mycelium and the spore concentration was determined using a Thoma chamber. To synchronize the germination, 2 × 10^5^ mL^–1^ spores were germinated in potato dextrose broth (PDB) at 24 g L^–1^ at room temperature for 3 h. Forty-four fully developed leaves were detached from four 6-week-old *Arabidopsis* plants (three leaves × plant), grown in a growth chamber maintained at 22°C and 70% relative humidity, with a 12 h/12 h day/night cycle (PAR level of 100 μmol m^–2^ s^–1^). The detached leaves were placed in square petri dishes with petioles embedded in 0.8% agar. Six droplets of spore suspension (5 μL each) were placed on the surface of each leaf. Mock inoculation was performed using PDB. Leaves were incubated at 24°C with a 12-h photoperiod.

### Visualization of *B. cinerea* Mycelium and PME Activity in *Arabidopsis* Leaves

After 72 h post *Botrytis cinerea* inoculation, *Arabidopsis* leaves were dipped in a solution with 0.05% (w/v) RR and subjected to vacuum infiltration for 10 min. Excess of dye was removed by several washes of stained leaves with water. Chlorophyll was extracted with hot 75% ethanol and leaves were mounted in 50% glycerol and examined by light microscopy using Nikon eclipse E200 microscope. Images were taken with a Nikon Digital Sight DS-Fi1c camera.

### Determination of PME and Pectinase Activity

Total protein extracts were obtained by homogenizing uninfected and infected *Arabidopsis* leaves in the presence of 1 M NaCl, 12.5 mM Citric Acid, 50 mM Na_2_HPO_4_, 0.02% Sodium Azide, protease inhibitor 1:100 v/v (P8849, Sigma), pH 6.5 (2 mL of extraction buffer per g of tissue). The homogenates were shaken for 1.30 h at 4°C, centrifuged at 15.000 × *g* for 15 min, and the supernatant collected. Protein concentration was determined in the supernatants using Bradford protein assay method (Bradford reagent, Sigma-Aldrich) and bovine serum albumin as standard (Bradford, 1976). After separation by sodium dodecyl sulfate–polyacrylamide gene electrophoresis (SDS-PAGE; Biorad), proteins were analyzed by Coomassie blue staining (SimplyBlue™ SafeStain, Invitrogen). PECTOPLATE was prepared with 0.1% (w/v) of apple pectin (molecular weight range 30,000–100,000 Da; 70–75% esterification; 76282, Sigma-Aldrich, St. Louis), 1% (w/v) SeaKem^®^ LE agarose (Lonza, Basel, Switzerland, Catalog no: 50004E), 12.5 mM citric acid and 50 mM Na_2_HPO_4_, pH 6.5. The gel was cast into 120 mm square petri dishes (50 mL per plate) and allowed to polymerize at room temperature. Wells with a diameter of 4 mm were obtained with a steel cork borer and equal amounts of protein samples (2 μg of total protein in 20 μL) were loaded in each well. Plates were incubated at 30°C for 16 h, and stained with 0.05% (w/v) RR (R2751; Sigma-Aldrich, St. Louis) for 30 min. The plates were de-stained by several washes with water and the area of the fuchsia-stained haloes, resulting from de-methylesterification of pectin and the area of inner unstained haloes, resulting from the hydrolysis of pectin in the gel, were measured with Image J software (Abramoff et al., 2004). Pectinase activity of *B. cinerea* was obtained by culturing the fungus in liquid Czapek Dox medium (2 g L^–1^ NaNO_3_, 1.0 g L^–1^ K_2_HPO_4_, 0.5 g L^–1^ MgSO_4_, 0.5 g L^–1^ KCl, 0.01 g L^–1^ FeSO_4_) containing 0.5% Polygalacturonic Acid (w/v; P3850, Sigma-Aldrich, St. Louis) as the sole carbon source. The flasks were inoculated with 1 mL of conidia (4 × 10^5^ conidia mL^-1^) and incubated on a rotary shaker at 100 *rpm* at 23°C for 3 days. *B. cinerea* PME activity was induced in the *B. cinerea* culture by adding 0.5% apple pectin (w/v; 76282, Sigma-Aldrich, St. Louis) and after 10 h the culture filtrate was collected. Recombinant AtPMEI-1 expressed in *Pichia pastoris* and purified to homogeneity (Raiola et al., 2004) was pre-incubated for 15 min with protein extracts before loading the mixture in the wells. Known amounts of commercially available PME from orange peel (P5400; Sigma-Aldrich, St. Louis) and of a Polygalacturonase (PG) from *Aspergillus japonicus* (P3304; Sigma-Aldrich, St. Louis) were used in PECTOPLATE. One PME unit is defined as the amount of enzyme required to release 1.0 microequivalent of acid from pectin per min. One PG units is defined as the amount of enzyme required to release 1.0 μmol of reducing sugar measured as D-Galacturonic acid from Polygalacturonic Acid per min. The area of the PME and PG haloes measured as above described were used to generate two standard curves, which was used to calculate the total PME and pectinase activity of the sample extracts.

### Oligogalacturonides Extraction and HPAEC-PAD Analysis

To avoid loading of RR dye on high-performance anion-exchange chromatography with pulsed amperometric detection (HPAEC-PAD), the circular pieces of gels corresponding to the area of inner clear haloes of 15 loaded wells were excised from the unstained plate, by comparison with a stained plate, and collected in 15 mL Falcon tubes. As control, a similar quantity of gel was excised from a zone far from the haloes and from an unloaded PECTOPLATE. Pectin fragments were extracted from the gels by adding 5 mL of water and incubating the samples at 30°C for 3 h in a water bath. The OGs degree of polymerization (DP) and their relative abundances in the samples were analyzed by HPAEC-PAD with an ICS-3000 apparatus (Dionex Corporation, Sunnyvale, CA, USA) equipped with a CarboPac PA-200 separation column (2 mm ID × 250 mm; Dionex Corporation) and a Carbopac PA-200 guard column (2 mm ID × 50 mm; Dionex Corporation). A flow of 0.4 mL/min was used and the temperature was kept at 25°C. The injected samples (25 μL) were separated using a linear gradient of 0.05 M KOH (solvent A) and 1 M KOAc in 0.05 M KOH (solvent B) using the following conditions: 0–31 min from 90% A to 20% A and from 10% B to 80% B. Before injection of each sample, the column was equilibrated with 90% A and 10% B for 10 min. Peaks were identified by comparison with OGs having known DPs (Trigalacturonic acid, T7407 from Sigma-Aldrich, St. Louis and Dodeca-galacturonic acid, available in the lab) and quantified using the Chromeleon chromatography software package (version 6.8; Dionex, Olten, Switzerland).

## Results and Discussion

### Ruthenium Red Allows Staining of Both *B. cinerea* Mycelium and PME Activity in *Arabidopsis* Leaf Tissue During Fungal Infection

**FIGURE 1:**
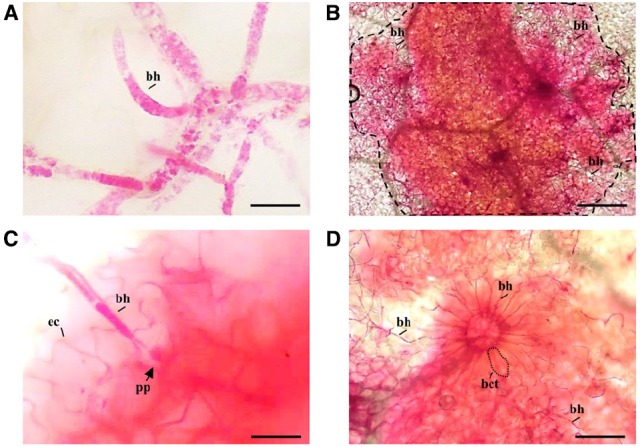
**Visualization of PME activity and *Botrytis cinerea* mycelium in infected WT *Arabidopsis* leaves during fungal infection. (A)** Ruthenium red staining of *B. cinerea* hyphae. Scale bar = 10 μm. **(B)** Picture representing an *Arabidopsis* leaf at 72 h post *Botrytis* infection stained with RR and observed with a light microscope. The presence of mycelium correlates with a strong accumulation of the dye at the level of lesion area (highlighted with a dotted line). Scale bar = 100 μm. **(C)** A magnification showing accumulation of PME activity close to fungal hyphae penetration. Scale bar = 25 μm. **(D)** *B. cinerea* penetrates on the surface of *Arabidopsis* leaves exploiting a passage obtained after detachment of a trichome. Scale bar = 100 μm. ec, *Arabidopsis* epidermal cell border; bh, *B. cinerea* hyphae; pp, point of penetration; bct, basal cell of a trichome.

Specific *Arabidopsis* PME isoforms are induced during *B. cinerea* infection in *Arabidopsis* leaves (Raiola et al., 2011; Lionetti et al., 2012). RR was used here to detect PME activity at histopathological level, in infected leaves following *B. cinerea* infection. Surprisingly, RR strongly stained *Botrytis* mycelium. The dye seems to accumulate in intracellular vesicular bodies, most likely staining fatty acid in lipid droplets and/or DNA in nuclei but the precise subcellular localization requires further studies (Figure 1A). The staining of the infected leaves with RR showed a strong fuchsia coloration restricted to the lesion areas where the mycelium mainly accumulates respect to a more clearer background observed far from the necrotized zones in the same tissue (Figure 1B). At the level of hypha penetration, the staining can be observed both in CW of epidermal cells like also in a more diffused and strong coloration, probably due to a sum of staining of the deeper multilayer of mesophyll cells (Figure 1C). This evidence indicates RR dye as a useful tool to simultaneously monitoring PME activity and growth of fungal hyphae in plant tissue and giving the opportunity to explore physiological phenomena. For example, in Figure 1D, fungal hyphae exploit the intercellular spaces of fuchsia stained basal cells of a detached trichome to penetrate in the leaf tissue.

### PECTOPLATE Provides a Simultaneous Phenotyping of PME and Pectinase Activities in Protein Extracts of Infected Plant Tissues and Their Potential in Generating Oligogalacturonides

**FIGURE 2:**
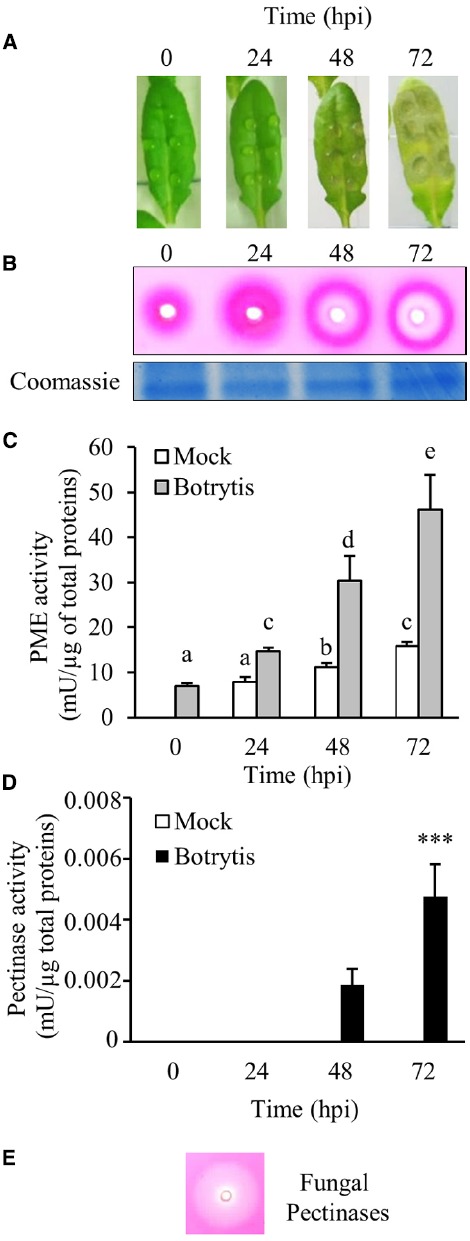
**PME activity in total protein extract of WT *Arabidopsis* plants during *Botrytis cinerea* infection. (A)** Leaves of *Arabidopsis* plants at different hours post *B. cinerea* inoculation. **(B)** Radial gel diffusion assay showing PME (fuchsia halo) and pectinase (inner clear halo) activities in proteins extracted from uninfected and infected leaves. Two μg of total proteins, quantified using Bradford protein assay method, were loaded in each well. Coomassie blue stained gel is shown as loading control. Quantification of **(C)** PME and **(D)** pectinase activity in protein extracts from uninfected and infected leaves. Data represent average ± standard deviation of a representative experiment (number of leaves = 6). The experiment was repeated three times with similar results. The different letters reported on the bars in C indicate data sets significantly different according to ANOVA followed by Tukey’s test (*P* < 0.05). Asterisks in D indicate significant differences in pectinase activity between 48 and 72 hpi according to Student’s *t*-test (***P < 0.001). **(E)** Clear halo produced by loading a polygalacturonase from *Aspergillus Japonicum*. Mock, protein extracts from mock-inoculated leaves; Botrytis, protein extracts from *Botrytis* inoculated leaves; mU, milliunits; hpi, hour post inoculation.

Total protein extracts isolated from *Arabidopsis* rosette leaves at different times after *B. cinerea* infection or after mock inoculation were assayed using PECTOPLATE (Figure 2). Wells obtained in a gel made with agarose-high methylesterified pectin were loaded with equal amounts of total proteins extracted from mock and *Botrytis*-inoculated *Arabidopsis* leaves (Figures 2A,B). De-methylesterification of pectin by PME produced in the tissues was quantified after RR staining by measuring the area of the fuchsia haloes. As previously reported, a PME activity was revealed in non-inoculated leaves (Figures 2B,C,; Lionetti et al., 2007). A PME activity was slightly induced in detached leaves at 48 up to 72 h after mock-inoculation (Figure 2C). This result is consistent with previous evidence indicating an induction of PME activity by rubbing the leaves with an abrasive powder in *Arabidopsis* (Lionetti et al., 2014). These data indicate that PME activity is involved in response to wounding in *Arabidopsis*, as already reported in banana and tobacco plants (von Dahl et al., 2006; Dorokhov et al., 2012; Ma et al., 2013). Upon *Botrytis* infection, the induction of PME activity was markedly higher respect to control and a steady increase was observed starting from 24 hours post inoculation (hpi) and persisting throughout all the period of infection analyzed (Figures 2B,C). Interestingly and unexpected, an inner clear halo appears within the fuchsia halo at 48 hpi and expands further at 72 hpi (Figures 2B,D). Similarly, *Aspergillus japonicum* PG (as well as an aliquot of cultural filtrate obtained from *B. cinerea* enriched in pectinase activity; data not shown) produced a clear halo when loaded in PECTOPLATE (Figure 2E) indicating that the inner clear halo observed before can be associated to pectinolytic activities secreted by *Botrytis* during infection.

**FIGURE 3:**
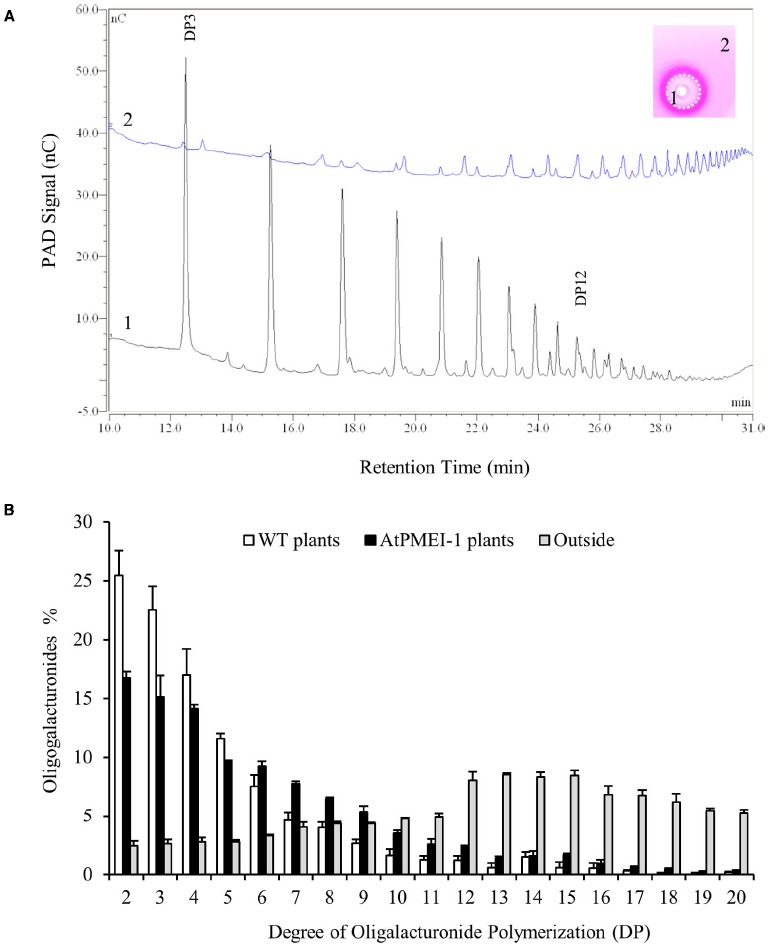
**Chromatographic profiles and quantification of OGs in PECTOPLATE (A) Oligogalacturonides released by pectinolytic activity expressed in WT plants during *Botrytis* infection relative to inner clear haloes (1) and relative to a zone of the plate far from the haloes (2) were analyzed by HPAEC-PAD using a CarboPac PA-200 anionic exchange column.** The degree of polymerization (DP) of OGs was recognized by comparing the retention times of the peaks in the chromatogram with of those of OGs with DP3 and DP 10. **(B)** The relative abundance of OGs released by pectinolytic activity expressed in WT and AtPMEI-1 plants at 72 h post *Botrytis* infection were quantified using the Chromeleon chromatography software. As control, pectin extracted from a point of the gel far from the haloes (outside) was also quantified. Data represent the average ± SD of a representative experiment (number of leaves = 3). The experiment was repeated three times with similar results.

The potential of pectin degradation associated to the enzymatic activities expressed in *Arabidopsis* tissue after 72 h after *Botrytis* infection was quantified. The portions of gel relative to inner clear haloes of 15 loaded samples were excised from an unstained PECTOPLATE, by comparison with the haloes in a stained plate. Pectin fragments were extracted from the gels and analyzed by HPAEC-PAD using a CarboPac PA-200 anionic exchange column (Figure 3A). The chromatographic profile relative to the inner clear haloes shows an enrichment of OGs with a low DP, indicating a pectin degradation. As control, the pectic fraction extracted from a zone of the plate far from the haloes was also analyzed and as expected, is characterized by quite undigested pectins. A similar chromatogram was observed when pectin extracted from unloaded PECTOPLATE was analyzed under the same conditions (data not shown). These results confirm that the inner clear halo is produced by pectinase activities. A quantification of the relative abundance of generated OGs (Figure 3B) indicate that OGs with a DP up to 5 represent the 76.48% in the inner clear haloes respect to the 10.57% in a gel area far from the haloes. Overall the results indicate that PECTOPLATE can be exploited to perform in one assay the phenotyping of the activities expressed from the pectinolytic machinery secreted in *Arabidopsis* during fungal infection and the characterization of their degradative effects on a specific pectic substrate.

**FIGURE 4:**
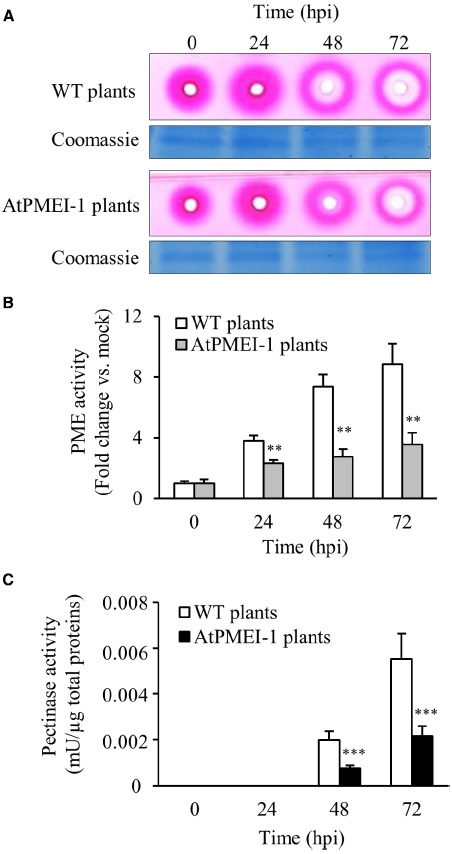
**Phenotyping of pectinolytic machineries expressed in WT and AtPMEI-1 plants during *Botrytis* infection. (A)** PECTOPLATE showing PME and pectinase activities expressed in WT and *Arabidopsis* AtPMEI-1 plants at different stages of *Botrytis* infection. **(B)** The PME and **(C)** pectinase activities were quantified at the indicated hours post inoculation. Two micrograms of total proteins, quantified using Bradford protein assay method, were loaded in each well. Coomassie blue stained gel is shown as loading control. Data represent average ± standard deviation of a representative experiment (number of leaves = 6). Asterisks indicate significant differences between WT and ATPMEI-1 plants according to Student’s *t-test* (**P < 0.01; ***P < 0.001). The experiment was repeated three times with similar results; mU, milliunits; hpi, hour post inoculation.

PECTOPLATE could be particularly useful for the phenotyping of plants with altered response to pathogens and/or on mutants with altered in CW structure or CW enzymes. Here, the method is applied to characterize transgenic plant overexpressing the PMEI AtPMEI-1 (AtPMEI-1 plants). These plants have a lower level of basal PME activity, an higher degree of pectin methylesterification and are more resistant to *Botrytis* respect to untransformed WT plants (Lionetti et al., 2007). Equal amounts of total protein extracts isolated from rosette leaves of WT plants and AtPMEI-1 plants at different times after *B. cinerea* infection or after mock inoculation were assayed using PECTOPLATE and PME and pectinase activity quantified (Figure 4). As reported before, the level of PME activity in transgenic plants was lower than in WT plants. Notably, a significantly lower level of PME activity was detected in ATPMEI-1 plant extracts during *Botrytis* infection respect to WT extracts (Figures 4A,B). Moreover, in AtPMEI-1 plant extracts a significant lower pectinase activity was observed at both 48 h and 72 hpi (Figure 4C). Consistently, the comparison of the OG profiles indicates an accumulation of OGs with a lower DP in WT respect to AtPMEI-1 plants (Figure 3B). These and previous results indicate that both basal and infection-induced PME activity is reduced in AtPMEI-1 plants, and that both reductions can modulate pectin degradation by *Botrytis* in *Arabidopsis*. These findings corroborate PECTOPLATE as useful tools to characterize pectinolytic machinery and to profile their degradation products in transgenic plants and mutants of interest.

### The Use of PMEIs in PECTOPLATE Discriminates Activities of Plant PMEs from Pathogen PMEs During Fungal Infection

**FIGURE 5:**
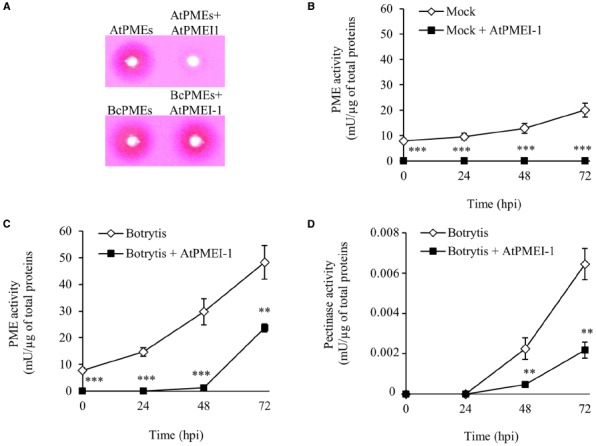
**Effect of exogenous addition of recombinant AtPMEI-1 on PME and pectinase activity. (A)** ATPMEI-1 expressed in *Pichia pastoris* and purified to homogeneity was exogenously added to the proteins extracted from WT leaves and to a culture filtrate of *B. cinerea* enriched in PME activity. Inhibitory effect of AtPMEI-1 on pectinase and PME activity in protein extracts from **(B)** mock treated and **(C,D)** *B. cinerea* inoculated *Arabidopsis* leaves. Data represent average ± standard deviation of a representative experiment (number of leaves = 3). Asterisks indicate significant differences between ATPMEI-1-treated and untreated extracts according to Student’s *t*-test (**P < 0.01; ***P < 0.001). The experiment was repeated three times with similar results. Mock, protein extracts from mock-inoculated leaves; Botrytis, protein extracts from *Botrytis* inoculated leaves; + AtPMEI-1, addition of AtPMEI-1 to the relative protein extract; mU, milliunits; hpi, hour post inoculation.

PME inhibitor isoforms up to now characterized are able to inhibit plant PMEs but not microbial PME secreted during infection (Raiola et al., 2004; Reca et al., 2012). The addition of the recombinant *Arabidopsis* AtPMEI-1, previously purified in *Pichia pastoris*, to a leaf protein extracts of an uninfected *Arabidopsis* completely inhibits resident PME activity (Figure 5A, upper panel). AtPMEI-1, added to a culture filtrate of *B. cinerea* enriched in PME activities is ineffective to inhibit fungal PMEs (Figure 5A, lower panel). The effect of AtPMEI-1 on the PME and pectinase activities secreted in mock and *Botrytis*-inoculated *Arabidopsis* leaves was tested. The exogenous addition of AtPMEI-1 to the proteins extracted from mock treated leaves completely inhibits the induced PME activity (Figure 5B). When added to the protein extracts obtained from *B. cinerea* infected leaves, AtPMEI-1 fully inhibits PME activity induced up to 24 hpi while an incomplete inhibition was observed on PME activity at 48 and 72 hpi, reaching 96% and 51% respectively (Figure 5C). Overall these results indicate that the PME activity induced at 24 h and 48 h is almost plant derived, while both fungal and plant activities are detectable mainly at the latter stage of infection analyzed. Moreover, the exogenous addition of AtPMEI-1 reduces pectin degradation by pectinase activity (Figure 5D) indicating that plant PME activity, in addition to fungal PME activity, favors the degradation of pectin carried out by *Botrytis* pectinases. Overall, these results indicate that PMEIs can be used in PECTOPLATE, as well as in other assay, as tools to discriminate between fungal and plant-derived PME activity.

## Conclusion and Outlooks

Progresses have been made in the isolation and characterization of CW and CW degrading enzymes involved in different physiological processes. However, very little is known about their dynamic changes occurring during plant–pathogen interactions. Tracking the activities of these proteins during pathogen infections will enhance our understanding of the involvement of CW polysaccharides and enzymes during plant defense. In this manuscript are presented different tools based on RR staining and applied to the field of plant–microbe interactions. Firstly, RR is proposed as useful dye to monitor at once both *B. cinerea* mycelium growth and induction of PME activity in plant tissue during fungal infection. Moreover, PECTOPLATE is presented as a simple, inexpensive and sensitive method for phenotyping in the same assay the activities of pectinolytic machinery expressed in infected plant tissues and its degradative potential. The spatial separation of the various CW enzymatic activities in the gel combined with the possibility to characterize their specific enzymatic products provides information that cannot be achieved in an “in liquid” enzymatic assay. Associated to mass spectrometry, PECTOPLATE can give a more detailed characterization on the enzyme involved and on the structure of pectic oligomers (Lionetti et al., 2007). Pectin with different degree and pattern of substitution (methylesterification, acetylesterification) could be used as substrate to understand the effect of pectin structure on pectinase activity and on the OGs release. Information about CW fragments released during infection could be obtained by using as possible alternative substrate in PECTOPLATE, CWs or CW fractions extracted from the tissue that will be infected. The same protein extracts could be also assayed for other CW degrading enzyme activity in a plate containing different specific CW substrate. For example, cellulose and Congo red staining can be used for the quantification of cellulase activity (Carder, 1986). The use of PMEIs in PECTOPLATE offers the possibility to discriminate between activities of plant PMEs from pathogen PMEs during fungal infection. Overall, these findings provide new tools that will helps to gain insights into CW mechanisms underlying different host–pathogen interactions.

### Conflict of Interest Statement

The author declares that the research was conducted in the absence of any commercial or financial relationships that could be construed as a potential conflict of interest.
